# Antioxidant and Protective Effect of Ethyl Acetate Extract of *Podophyllum Hexandrum* Rhizome on Carbon Tetrachloride Induced Rat Liver Injury

**DOI:** 10.1155/2011/238020

**Published:** 2011-01-09

**Authors:** Showkat Ahmad Ganie, Ehtishamul Haq, Akbar Masood, Abid Hamid, Mohmmad Afzal Zargar

**Affiliations:** ^1^Department of Biochemistry, University of Kashmir, Srinagar 190006, India; ^2^Department of Biotechnology, University of Kashmir, Srinagar 190006, India; ^3^Indian Institute of Integrative Medicine, Jammu (Council of Scientific and Industrial Research), India

## Abstract

The antioxidant and hepatoprotective activities of ethyl acetate extract was carefully investigated by the methods of DPPH radical scavenging activity, Hydroxyl radical scavenging activity, Superoxide radical scavenging activity, Hydrogen peroxide radical scavenging activity and its Reducing power ability. All these *in vitro* antioxidant activities were concentration dependent which were compared with standard antioxidants such as BHT, *α*-tocopherol. The hepatoprotective potential of *Podophyllum hexandrum* extract was also evaluated in male Wistar rats against carbon tetrachloride (CCl_4_)-induced liver damage. Pre-treated rats were given ethyl acetate extract at 20, 30 and 50 mg/kg dose prior to CCl_4_ administration (1 ml/kg, 1:1 in olive oil). Rats pre-treated with *Podophyllum hexandrum* extract remarkably prevented the elevation of serum AST, ALT, LDH and liver lipid peroxides in CCl_4_-treated rats. Hepatic glutathione levels were significantly increased by the treatment with the extract in all the experimental groups. The extract at the tested doses also restored the levels of liver homogenate enzymes (glutathione peroxidase, glutathione reductase, superoxide dismutase and glutathione-S- transferase) significantly. This study suggests that ethyl acetate extract of *P. hexandrum* has a liver protective effect against CCl_4_-induced hepatotoxicity and possess *in vitro* antioxidant activities.

## 1. Introduction

The partially reduced metabolites of oxygen and nitrogen, commonly referred to as free radicals are highly toxic and reactive. They have been postulated to be increased in majority of diseases like aging, atherosclerosis, cancer, diabetes, liver cirrhosis, cardiovascular disorders, and so forth, [1, 2]. The most common reactive oxygen species are superoxide anion (O_2_·-), hydrogen peroxide (H_2_O_2_), peroxyl radical (ROO^●^), and highly reactive hydroxyl radical (OH^●^). Oxidative processes are the most important routes for producing free radicals in living systems. The liver, because of its strategic anatomical action and its large capacity for metabolic conversions, is exposed to many kinds of xenobiotics and therapeutic agents. Due to these facts, efforts to find suitable curatative agents for treatment of liver diseases in natural products of plant and mineral origin are being made [2]. Liver injury induced by CCl_4_ is the best characterized system of xenobiotics-induced hepatotoxicity in living system. It is a volatile organic chemical and causes liver, kidney and lung damage through free radical mediated process.

Antioxidants are the substances that when present in low concentration significantly delay or reduce the oxidation of the substrate [3]. Antioxidants protect the body from damaging oxidation reactions by reacting with free radicals and other reactive oxygen species; therefore, diseases linked with free radicals can be prevented by antioxidant therapy. The subject has gained an immense importance and current research trends are directed towards finding naturally occurring antioxidants particularly of plant origin [4–6]. Currently, available synthetic antioxidants like butylated hydroxy anisole (BHA), butylated hydroxy toluene (BHT), tertiary butylated hydroquinone, and gallic acid esters have been suspected to cause negative health effects. Hence, strong restrictions have been placed on their application, and there is a trend to substitute them with naturally occurring antioxidants. Moreover, these synthetic antioxidants also show low solubility and moderate antioxidant activity [7]. BHA and BHT are suspected of being responsible for liver toxicity and carcinogenesis [8, 9]. Traditionally used natural antioxidants from tea, wine, fruits, vegetables, spices, and medicine (e.g., rosemary and sage) are already exploited commercially either as antioxidant additives or a nutritional supplements [10]. Also, many other plant species have been investigated in search of novel antioxidants [11–14], but generally, there is still a demand to find more information concerning the antioxidant potential of plant species. It has been mentioned that the antioxidant activity of plants might be due to their phenolic compounds [15]. Flavonoids are a group of polyphenolic compounds with known properties which include free-radical scavenging, inhibition of hydrolytic and oxidative enzymes, and anti-inflammatory actions [16–18]. The use of traditional medicine is widespread, and plants still present a large source of natural antioxidants that might serve as leads for the development of novel drugs.

The evaluation of plant extracts antioxidant capacity is not an easy task, since many different methods can be used to determine this activity. Substrates, conditions, methods, and concentrations can also affect the estimated activity [19]. Rhizome powder of *P. hexandrum* is locally used as a laxative, to treat intestinal parasites, worms, warts, and tumourous growth on skin [5]. The current study was carried out to examine the antioxidant and protective effects of ethyl acetate rhizome extract of *P. hexandrum* in the rat model of CCl_4_-induced liver injury.

## 2. Materials and Methods

### 2.1. Plant Material Collection and Extraction

The rhizome of *P. hexandrum* was collected from higher reaches of Aharbal, Shopian, J&K, India, in the month of May and June 2009, identified by the Centre of Plant Taxonomy, Department of Botany, University of Kashmir, and authenticated by Dr. Irshad Ahmad Nawchoo (Department of Botany) and Akhter Hussain Malik (Curator, Centre for Plant Taxonomy, University of Kashmir). A reference specimen has been retained in the herbarium of the Department of Botany at the University of Kashmir under reference number KASH-bot/Ku/PH-702-SAG. 

The plant material (rhizome) was dried in the shade at 30 ± 2°C. The dried rhizome material was ground into a powder using mortar and pestle and passed through a sieve of 0.3 mm mesh size. The powder obtained was extracted with ethyl acetate using a Soxhlet extractor (60–80°C). The extract was then concentrated with the help of rotary evaporator under reduced pressure and the solid extract was stored in refrigerator for further use.

### 2.2. Animals

Adult male albino rats of Wistar strain weighing 200–250 g used throughout this study were purchased from the Indian Institute of Integrative and Medicine Jammu (IIIM). The animals had access to food and water *ad libitum*. The animals were maintained in a controlled environment under standard conditions of temperature and humidity with an alternating 12 hr light and dark cycle. The animals were maintained in accordance with the guidelines prescribed by the National Institute of Nutrition, Indian Council of Medical Research, and the study was approved by the Ethical Committee of the University of Kashmir.

## 3. Experimental Methods

### 3.1. DPPH Radical Scavenging Activity

The 1, 1- diphenyl-2- picryl- hydrazyl (DPPH) assay was performed by using the method of [4, 20]. Various concentrations of plant extract (100–1000 *μ*g/mL were added to 1ml of the 0.004% methanol solution of DPPH, and the mixture was vortexed vigorously. The tubes were then incubated at room temperature for 30 minutes in dark, and the absorbance was taken at 517 nm. Lower absorbance of the reaction mixture indicates higher free radical scavenging activity. Alpha tocopherol and BHT was taken as known free-radical scavengers. Percentage inhibition activity was calculated by.

(1) $$\%\;\text{inhibition}=\left[\frac{\left({\text{A}}_{0}-{\text{A}}_{1}\right)}{{\text{A}}_{0}}\right]\times 100,$$

where A_0_ was the absorbance of the control and A_1_ was absorbance in the presence of *P. hexandrum* extract/known antioxidant.

### 3.2. Assessment of Hydroxyl Radical Scavenging Property

Hydroxyl radical, generated from the Fe^3+^-Ascorbate-H_2_O_2_ (Fenton reaction), was evaluated by degradation of deoxyribose that produced thiobarbituric acid reactive species (TBARS) [21]. The reaction mixture contained 25 mM deoxyribose, 10 mM Ferric chloride, 100 mM ascorbic acid, 2.8 mM H_2_O_2_ in 10 mM KH_2_PO4 (pH 7.4), and various concentrations of *P. hexandrum* rhizome ethyl acetate extract. The reaction mixture was incubated at 37°C for 1 h. Then, 1 mL of 1% thiobarbituric acid and 1 mL of 3% trichloroacetic acid was added and mixture heated at 100°C for 20 min. The TBARS was measured spectrophotometrically by taking absorbance at 532 nm. The results were expressed as percentage inhibition of deoxyribose oxidation, as determined by.

(2) $$\text{Percentage}\;\text{inhibition}=\left[\frac{\left(\text{A}-\text{B}\right)}{\text{A}}\right]\times 100,$$

where A was the malondialdehyde produced by Fenton reaction treated alone, and B was the malondialdehyde produced in the presence of *P. hexandrum* extract/known antioxidant.

### 3.3. Assessment of Superoxide Anion Radical Scavenging Property

Superoxide anion radical generated by the Xanthine/Xanthine oxidase system was spectrophotometrically determined by monitoring the product of nitroblue tetrazolium (NBT) using the method of [22]. A reaction mixture containing 1.0 mL of 0.05 M phosphate buffer (pH 7.4), 0.04 mL of 0.15% BSA, 0.04 mL of 15.0 mM NBT, and various concentrations of (plant extract and known antioxidant) was incubated at 25°C for 10 min, and the reaction was then started by adding 0.04 mL of 1.5 U/mL Xanthine oxidase and again incubated at 25°C for 20 min. The absorbance of the reaction mixture was measured at 560 nm. Decreased absorbance of the reaction mixture indicates increased superoxide anion radical scavenging activity.

The scavenging activity of the plant extract on superoxide anion radical was expressed as

(3) $$\;\%\;\text{inhibition}=\left[\frac{\left({\text{A}}_{0}-{\text{A}}_{1}\right)}{{\text{A}}_{0}}\right]\times 100,$$

where A_0_ was the absorbance of the control and A_1_ was absorbance in the presence of *P. hexandrum* extract/known antioxidant.

### 3.4. Assessment of Hydrogen Peroxide Scavenging Activity

The ability of *P. hexandrum* ethyl acetate extract to scavenge hydrogen peroxide was evaluated according to the method of [23, 24]. A solution of H_2_O_2_ (2 mmole) was prepared in phosphate buffer (pH 7.5). Plant extract (50–300 *μ*g/mL) were added to the hydrogen peroxide solution (0.6 mL). Absorbance of hydrogen peroxide at 230 nm was determined after 15 minutes against a blank solution containing phosphate buffer without hydrogen peroxide. BHT was taken as known standard. The scavenging activity of the plant extract on H_2_O_2_ was expressed as

(4) $$\text{\%}\;\text{scavenged}\;\left[{\text{H}}_{2}{\text{O}}_{2}\right]=\left[\frac{{\text{A}}_{0}-{\text{A}}_{1}}{{\text{A}}_{0}}\right]\times 100,$$

where A_0_ is the absorbance of the control and A_1_ is absorbance in the presence of plant extract and known standard.

### 3.5. Reducing Power

The reducing power of *P. hexandrum* rhizome extract was evaluated according to [25]. Different concentrations of the plant extract were mixed with 2.5 mL of 0.2 M phosphate buffer (pH 6.6) and 2.5 mL of 1% potassium hexacyanoferrate II. The mixture was incubated at 50°C for 20 minutes, 2.5 mL of 10% TCA was added to the mixture and centrifuged at 3000 rpm for 10 minutes. The upper layer of the solution (2.5 mL) was mixed with distilled water (2.5 mL) and FeCl_3_ (0.5 mL, 0.1%), and the absorbance was measured at 700 nm. BHT (butylated hydroxyl toluene) was taken as the known standard. Increased absorbance of the reaction mixture indicates stronger reducing power.

### 3.6. Dosage and Treatment

Rats were divided into six groups each containing seven rats. The plant extract was employed at oral doses of 20, 30, and 50 mg/kg-day. The extract was suspended in normal saline such that the final volume of extract at each dose was 1 mL which was fed to rats by gavage.

Group I: received olive oil vehicle only at 5 mL/kg-day.
Group II: received CCl_4_ in olive oil only.
Group III: were administered with vitamin E (50 mg/kg-day).
Group IV: received 20 mg/kg-day extract orally for fifteen days.
Group V: received 30 mg/kg-day extract orally for fifteen days.
Group VI: received 50 mg/kg-day orally for fifteen days.

On the thirteenth day, animals from groups II–VI were injected intraperitoneally with CCl_4_ in olive oil vehicle at a dosage of 1 mL/kg bw. The rats were sacrificed, 48 hr after CCl_4_ administration, and livers were collected; post mitochondrial supernatant of the liver tissue was prepared as written under preparation of PMS in Section 3.8.

### 3.7. Blood Collection for Estimation of AST, ALT, and LDH

Before sacrificing the experimental animals, blood was collected from retro-orbital plexus without the use of anticoagulant. The blood was allowed to stand for 10 min before being centrifuged at 5,000 g for 10 min to obtain serum for analysis of alanine aminotransferase (ALT), aspartate aminotransferase (AST), and serum lactate dehydrogenase (LDH). 

#### 3.7.1. Serum Alanine Aminotransaminase (ALT)

ALT was estimated by the method of Reitman and Frankel [26]. Briefly, 0.5 mL of substrate (2 mM *α*-ketoglutarate, 0.2 M DL-alanine in phosphate buffer 0.1 M pH 7.4) was incubated at 37°C for 5 minutes. 0.1 mL of freshly prepared serum will be added to the aliquot and again incubated at 37°C for 30 minutes. At the end of incubation 0.5 mL of 2, 4-dinitrophenylhydrazine was added, and the aliquot left for 30 minutes at room temperature. 0.5 mL of 0.4 N NaOH was added, and the aliquot is again left for 30 minutes. Absorbance was then recorded at 505 nm against water blank.

#### 3.7.2. Serum Aspartate Aminotransaminase (AST)

AST was again estimated by the method of Reitman and Frankel [26]. The substrate, however, was 2 mM *α*-ketoglutarate, 0.2 M DL-aspartate, and the rest of the procedure was same.

#### 3.7.3. Serum Lactate Dehydrogenase (LDH)

LDH was assayed according to the method of King [27]. To 1.0 mL of buffered substrate (sodium pyruvate 37.5 mM in phosphate buffer 100 mM, pH 7.4), 0.1 mL of freshly prepared serum was added, and the tubes were incubated at 37°C for 15 minutes. After adding 0.2 mL of NAD^+^ solution (10 mg/mL in phosphate buffer), the incubation was continued for another 15 minutes. The reaction was arrested by adding 0.1 mL of 2, 4-dinitrophenylhydrazine (0.02% in concentrated HCl), and the tubes were incubated again at 37°C for a further period of 15 min, after which 7.0 mL of 0.4 N NaOH was added, and the color developed was measured spectrophotometrically at 420 nm against a blank containing phosphate buffer only.

### 3.8. Preparation of Post Mitochondrial Supernatant (PMS)

Liver tissue was washed in ice-cold 1.15% KCl and homogenized in a homogenizing buffer (50 mM Tris-HCl, 1.15% KCl pH 7.4) using a Teflon homogenizer. The homogenate was centrifuged at 9,000 g for 20 minutes to remove debris. The supernatant was further centrifuged at 15,000 g for 20 minutes at 4°C to get PMS, subsequently used for various biochemical assays. Protein concentration was estimated by the method of Lowry et al. [28].

### 3.9. Estimation of Lipid Peroxidation (PMS)

Lipid peroxidation in liver tissue homogenate was estimated by the formation of thiobarbituric acid reactive substances (TBARS) by the method of [29]. In brief, 0.1 mL of tissue homogenate (PMS; Tris-HCl buffer, pH 7.5) was treated with 2 mL of (1 : 1 : 1 ratio) TBA-TCA-HCl reagent (0.37% thiobarbituric acid, 0.25 N HCl, and 15% TCA), placed in boiling water bath for 15 min, cooled, and centrifuged at room temperature for 10 min. The absorbance of the clear supernatant was measured against reference blank at 535 nm.

### 3.10. Determination of Total Sulphydryl Groups

The acid soluble sulphydryl groups (nonprotein thiols of which more than 93% is reduced glutathione (GSH) forms a yellow colored complex with DTNB that shows the absorption maximum at 412 nm. The assay procedure followed was that of [30]. 500 *μ*L of homogenate precipitated with 100 *μ*L of 25% TCA was subjected to centrifugation at 300 xg for 10 minutes to settle the precipitate. 100 *μ*L of the supernatant was taken in a test tube containing the 2 mL of 0.6 mM DTNB and 0.9 mL of 0.2 mM sodium phosphate buffer (pH 7.4). The yellow color obtained was measured at 412 nm against the reagent blank which contained 100 *μ*L of 25% TCA in place of the supernatant. Sulphydryl content was calculated using the DTNB molar extension coefficient of 13,100.

### 3.11. Glutathione Peroxidase (GPx)

GPx activity was assayed using the method of [31]. The assay mixture consisted of 1.49 mL of sodium phosphate buffer (0.1 M pH 7.4), 0.1 mL EDTA (1 mM), 0.1 mL sodium azide (1 mM), 0.1 mL 1 mM GSH, 0.1 mL of NADPH (0.02 mM), 0.01 mL of 1 mM H_2_O_2_, and 0.1 mL PMS in a total volume of 2 mL. Oxidation of NADPH was recorded spectrophotometrically at 340 nm and the enzyme activity was calculated as nmoles NADPH oxidized/min/mg of protein, using extinction coefficient of 6.22 × 10^3^ M^−1^ cm^−1^.

### 3.12. Glutathione Reductase Activity (GR)

GR activity was assayed by the method of [31, 32]. The assay mixture consisted of 1.6 mL of sodium phosphate buffer (0.1 M pH 7.4), 0.1 mL EDTA (1 mM), 0.1 mL 1 mM oxidized glutathione, 0.1 mL of NADPH (0.02 mM), 0.01 mL of 1 mM H_2_O_2_, and 0.1 mL PMS in a total volume of 2 mL. The enzyme activity measured as absorbance at 340 nm was calculated as nmoles of NADPH oxidized/min/mg of protein using extinction coefficient of 6.22 × 10^3^ M^−1^ cm^−1^.

### 3.13. Glutathione-S-Transferase (GST) Activity

GST activity was assayed using the method of [33]. The reaction mixture consisted of 1.67 mL sodium phosphate buffer (0.1 M pH 6.5), 0.2 mL of 1 mM GSH, 0.025 mL of 1 mM CDNB, and 0.1 mL of post mitochondrial supernatant in a total volume of 2 mL. The change in absorbance was recorded at 340 nm and the enzyme activity was calculated as nmoles of CDNB conjugates formed/min/mg protein using extinction coefficient of 9.6 × 10^3^ M^−1^ cm^−1^.

### 3.14. Superoxide Dismutase Activity (SOD)

SOD activity was estimated by Beauchamp and Fridovich [34]. The reaction mixture consisted of 0.5 mL of hepatic PMS, 1 mL of 50 mM sodium carbonate, 0.4 mL of 25 *μ*M NBT, and 0.2 mL of 0.1 mM EDTA. The reaction was initiated by addition of 0.4 mL of 1 mM hydroxylamine-hydrochloride. The change in absorbance was recorded at 560 nm. The control was simultaneously run without tissue homogenate. Units of SOD activity were expressed as the amount of enzyme required to inhibit the reduction of NBT by 50%.

### 3.15. Statistical Analysis

The values are expressed as mean ± standard deviation (SD). The results were evaluated by using the SPSS (version 12.0) and Origen 6 software and evaluated by one-way ANOVA followed by Bonferroni *t*-test. Statistical significance was considered when value of *P* was <.5.

## 4. Results

In this present study, the antioxidant activity of the ethyl acetate extract of the rhizome of *P. hexandrum* was investigated by using DPPH scavenging assay, hydroxyl assay, H_2_O_2_ assay, superoxide assay, and reducing power. Under *in vivo* situations, the extract was examined by determining the antioxidant enzymes activities and glutathione levels in the liver tissue homogenate. All the methods have proven the effectiveness of the ethyl acetate extract compared to known antioxidants BHT and *α*-tocopherol. 

### 4.1. DPPH Radical Scavenging Activity

**Figure 1:**
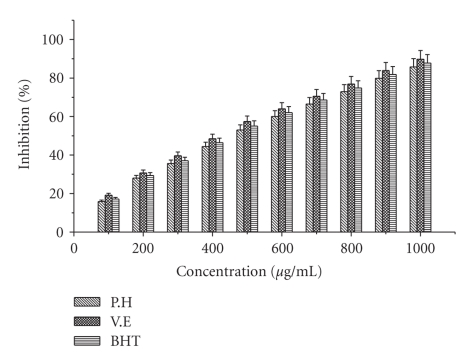
Effect of ethyl acetate extract and known antioxidants on DPPH radical scavenging activity. The results represent mean ± S.D. of 3 separate experiments. Absorbance at 517 nm.

Comparison of the antioxidant activity of the extract and standard antioxidants by DPPH method is shown in Figure 1. The ethyl acetate extract of *P. hexandrum* exhibited dose-dependent inhibition of DPPH activity, and the scavenging activities of the extract and known antioxidants increased with increasing concentration. At higher concentration (1000 *μ*g/mL), the extract and known antioxidants gave the highest percentage activities, that is, (85.77%), (89.77) and (87.7%), respectively. The percentage inhibition observed is in the following descending order *α*-tocopherol > BHT > ethyl acetate extract. These results suggest that ethyl acetate extract of *P. hexandrum* has a noticeable effect on scavenging of DPPH radical.

### 4.2. Hydroxyl Radical Scavenging Activity

**Figure 2:**
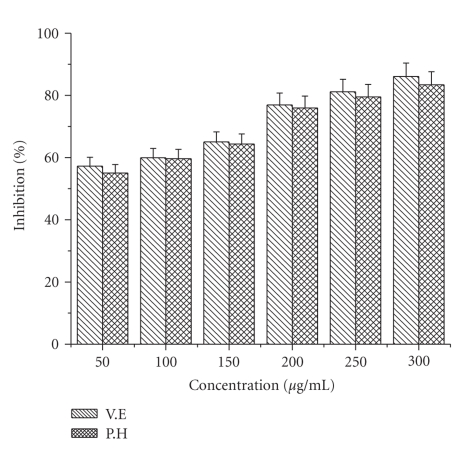
Effect of ethyl acetate extract and known antioxidants on hydroxyl radical scavenging activity. The results represent (mean ± S.D.) of 3 separate experiments. Results are reported as the percentage of the maximum formation of OH^●^ radical (100% deoxyribose oxidized): in absorbency, 100% is 1.540 ± 0.042 (control). Absorbance at 532 nm.

The effect of ethyl acetate extract of *P. hexandrum *on the inhibition of hydroxyl radical production was assessed by the iron (II)-dependent deoxyribose damage assay.  Figure 2 presents the results of the effects of examined ethyl acetate extract as well as known antioxidants on OH**^●^** radical production. The percentage of hydroxyl radical scavenging activity increased with the increasing concentration of extract and known antioxidants. At a concentration of 300 *μ*g/mL, the extract shows the maximum inhibitory effect of about 83.44% which was comparable to that of *α*-tocopherol (88 and 86%). The scavenging activity was found in the following order *α*-tocopherol > ethyl acetate extract.

### 4.3. Superoxide Anion Scavenging Activity

**Figure 3:**
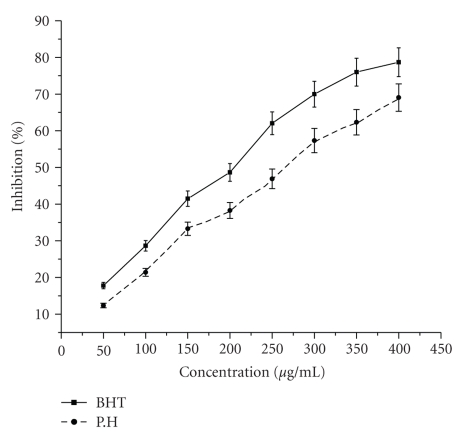
Effect of ethyl acetate extract and known antioxidant BHT on superoxide anion radical scavenging activity. The results represent mean ± S.D. of 3 separate experiments. Absorbance at 560 nm (Absorbance of control = 0.883 ± 0.23).

Results of superoxide anion scavenging activity of ethyl acetate extract of rhizome of *P. hexandrum* and known antioxidant BHT are shown in Figure 3. The extract and the standard demonstrate a dose response inhibition on superoxide anion radical. The % inhibition of superoxide generation by ethyl acetate extract and BHT was found to be 70.5% and 78.4% at a higher concentration of 400 *μ*g/mL. the results suggest that ethyl acetate extract has a potent superoxide scavenging effects.

### 4.4. Hydrogen Peroxide Scavenging Activity

**Figure 4:**
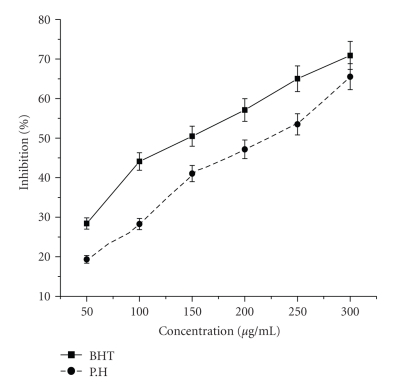
Effect of ethyl acetate extract and known antioxidant BHT on hydrogen peroxide radical scavenging activity. The results represent mean ± S.D. of 3 separate experiments. Absorbance at 230 nm (Absorbance of control = 0.653 ± 0.16).

As shown in Figure 4, the ethyl acetate extract of *P. hexandrum* also demonstrated hydrogen peroxide decomposition activity in a concentration-dependent manner. At the higher concentration (300 *μ*g/mL) of extract and BHT the H_2_O_2_ scavenging activity was found as 65% and 70%, respectively. These results showed that ethyl acetate extract had effective H_2_O_2_ scavenging activity.

### 4.5. Reducing Power Assay

**Figure 5:**
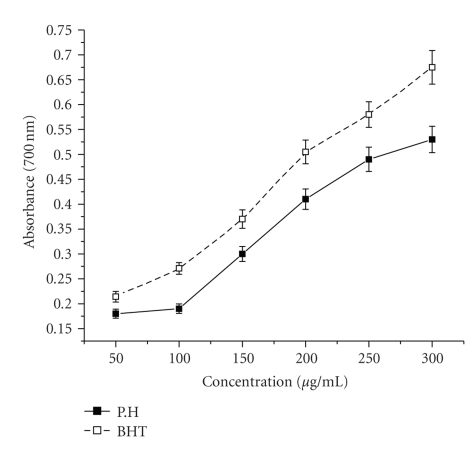
Effect of ethyl acetate extract and known antioxidant BHT on reducing power method. The results represent mean ± S.D. of 3 separate experiments. Absorbance at 700 nm.

Figure 5 shows the reductive capabilities of ethyl acetate extract of *P. hexandrum* when compared to the standard, BHT. Like the antioxidant activity, the reducing power increased with increasing amount of the extract. For the measurement of the reductive ability, the Fe^3+^-Fe^2+^ transformation was investigated in presence of the extract. Presence of reductants causes the reduction of the Fe^3+^/ferricyanide complex to the Fe^2+^ form. This Fe^2+^ can be monitored by measuring the formation of Perl's Prussian blue at 700 nm [35]. At higher concentration of plant extract (300 *μ*g/mL), reducing power value was around 0.53, while at the same concentration of BHT, the positive control used in this test, had a reducing power value of 0.65.

### 4.6. Biochemical Parameters

#### 4.6.1. Effect of Extract on Hepatic Markers

As depicted in Table 1, administration of CCl_4_ induced a marked increase in the levels of AST, ALT, and LDH levels as compared to the control group. Group I: (this group was given neither CCl_4_ nor treatment). They had normal values of AST (37.98), ALT (28.06), and LDH (89.68) units/mL. Group II: (the animals were given only CCl_4_). These rats were found to possess high values of AST (118.39), ALT (94.41), and LDH (218.62) units/mL. Group III: (treated with standard vitamin E + CCl_4_). There was a large fall in the values of AST (67.55), ALT (66.58), and LDH (170.88) units/mL. Group IV: (treated with 20 mg/kg bw ethyl extract + CCl_4_). There was a slight fall in the values of AST (100.41), ALT (79.33) and LDH (193.23) units/mL. Group V: (treated 30 mg/kg bw extract + CCl_4_). There was a decrease in the values of AST (65.37), ALT (67.22) and LDH (178.10) units/mL Group VI: (treated with 50 mg/kg bw extract + CCl_4_). There was a drastic decrease in the values of AST (54.16), ALT (60.03), and LDH (168.10). Group comparison between Group III, Group V and Group VI shows no significant variation in these parameters indicating that ethyl acetate extract has got the same effect as that of the vitamin E which was used as the positive control in this study.

#### 4.6.2. Effect of Extract on GSH and Antioxidant Enzyme Activities

The findings of present investigation indicate the ethyl acetate extract of *P. hexandrum* and modulate the liver against oxidative stress in a dose-dependent manner without any adverse effect on the animals at the dose levels (20, 30, and 50 mg/kg body weight/day for 15 days, respectively).  Table 2 shows oxidative stress by CCl_4_ caused significant alterations in hepatic antioxidant defense system like GSH, GR, GPx, SOD, and GST contents in comparison to the normal controls.

#### 4.6.3. Effect of Extract on Glutathione Reductase (GR)

Glutathione reductase (GR) activities in the liver homogenate of rats for all experimental groups are shown in the Table 2. The GR activity in the liver tissue homogenates of CCl_4_-treated rats was considerably lower than that of normal control rats (32.59 ± 2.10). In the pretreated group, which got the ethyl acetate extract for 15 days prior to CCl_4_, GR activity was significantly higher compared to the CCl_4_-treated group (2.30 ± 0.30). The GR activity in group III rats receiving vitamin E (25.44 ± 3.06) was found comparable to the group VI rats receiving the high concentration of plant extract (50 mg/kg bw/day) (24.55 ± 1.22). Rats of groups IV and V receiving the oral dose of 20 and 30 mg/kg bw/day plant extract increases the GR activity to 15.36 ± 1.47 and 19.65 ± 1 from 2.30 ± 0.3 (group II) Table 2.

#### 4.6.4. Effect of Extract on Glutathione Peroxidase (GP_X_)

CCl_4_ treatment caused a significant decrease in the level of glutathione peroxidase (GPx) activity in liver tissue when compared with control group (Table 2). The treatment of *P. hexandrum* ethyl acetate extract at the doses of 20, 30, and 50 mg/kg bw/day resulted in a dose-dependent increase of GP_X_ when compared to CCl_4_-treated rats. The liver of vitamin E-treated animals also showed a significant increase in GP_X_ activity compared to CCl_4_-treated rats.

#### 4.6.5. Effect of Extract on Superoxide Dismutase Activity (SOD)

The activities of SOD in the tissue homogenates of all experimental rats are shown in Table 2. In the liver homogenate, CCl_4_-treatment caused reduction of the SOD activity (17.16 units/mg protein) compared to the normal control group (35.41 units/mg protein). Enhancement of SOD activity was observed in case of 15 days treatment of ethyl acetate extract at the dose level of 20, 30, and 50 mg/kg body weight/day prior to the CCl_4_ administration, and the activity was found to be increased in a dose-dependent manner. Similar results were obtained with the antioxidant vitamin E.

#### 4.6.6. Effect of Extract on Glutathione-S-Transferase Activity (GST)

Table 2 showed significant decreased in hepatic glutathione-S-transferase (GST) activity upon CCl_4_ administration by 4.98 ± 0.49 as compared to the control group 15.22 ± 0.69. A significant increase in GST activity was observed in the group of rats treated with ethyl acetate extract of *P. hexandrum *at the oral dose of 20, 30, and 50 mg/kg bw/day by 7.60, 9.95 and 12.70, respectively, as compared to CCl_4_-treated group. In group III, the rats continuously treated with vitamin E (50 mg/kg bw/day) increased the GST activity significantly by 11.94 as compared to CCl_4_-treated group.

#### 4.6.7. Effect of Extract on Glutathione Levels (GSH)

Effect of ethyl acetate extract of *P. hexandrum* on GSH level for all experimental groups is shown in Table 2. CCl_4_ treatment caused significant decrease of GSH level in liver tissue homogenates compared to the normal group. When rats were treated by CCl_4_, GSH decreased from 103.94 ± 6.03 mg/g protein (control group) to 25.14 ± 2.59 mg/g protein (CCl_4_-treated group). Pretreatment of ethyl acetate extract for 15 days at the oral doses of 20, 30, and 50 mg/kg bw/day followed by 2 days of CCl_4_ treatment enhance the level of GSH to 56.65, 77.60, and 85.24 mg/g protein. Similar results were obtained with vitamin E.

#### 4.6.8. Effect of Extract on Lipid Peroxidation (LP)

**Figure 6:**
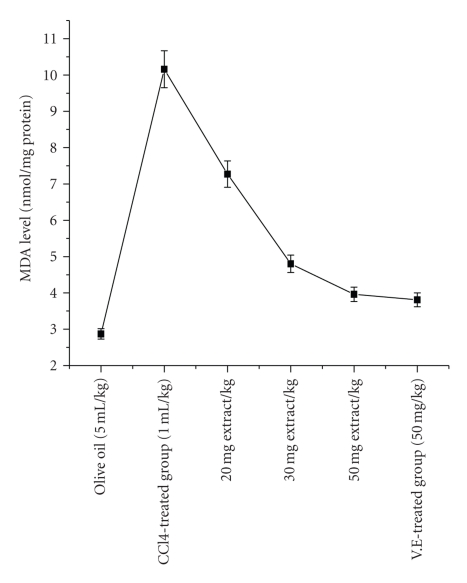
Effect of *Podophyllum hexandrum* ethyl acetate extract and vitamin E on liver homogenate lipid peroxidation of CCl_4_-treated rats *in vivo*. The results represent mean ± S.D. of 6 animals in each group and evaluated by one-way ANOVA followed by the Bonferroni *t*-test. Differences were considered to be statistically significant if *P* < .05.

The localization of radical formation resulting in lipid peroxidation, measured as MDA in rat liver homogenate is shown in Figure 6. From our results, it was found that *P. hexandrum* ethyl acetate extract could significantly decrease the formation of Malondialdehyde (MDA) in CCl_4_-treated rats. After CCl_4_ administration, the liver MDA level significantly increased from 2.87 ± 0.36 nmol/mg protein (control group) to 10.16 ± 0.95 nmol/mg protein. However, oral administration of *P. hexandrum* extract at the concentration of 20, 30, and 50 mg/kg bw/day, the liver MDA level decreased to 7.27 ± 0.26, 4.80 ± 0.32, and 3.73 ± 0.38, respectively. At the same time, the effect of vitamin E (50 mg/kg/day) on MDA levels in CCl_4_-treated rats was found to be reduced to 3.81 nmol/mg protein.

## 5. Discussion

Free radical oxidative stress has been implicated in the pathogenesis of a wide variety of clinical disorders, resulting usually from deficient natural antioxidant defenses. Potential antioxidant therapy, therefore, should include either natural free-radical scavenging antioxidant enzymes or agents which are capable of augmenting the activity of these enzymes. Reactive oxygen species (ROS) has received considerable attention in the recent past because of its role in several pathological conditions including cancer, diabetes, arthritis, aging, and atherosclerosis. ROS produced *in vivo *O_2_^−^, hydrogen peroxide (H_2_O_2_), and hypochlorous acid (HOCl), and H_2_O_2_ can interact in the presence of transition metal ions to yield a highly reactive oxidizing species, the hydroxy radical [36]. If human disease is believed to be due to the imbalance between oxidative stress and antioxidative defense, it is possible to limit oxidative tissue damage and hence prevent disease progression by antioxidant defense supplements. 

In our study, antioxidant effects of rhizome ethyl acetate extract of *P. hexandrum* were measured through a variety of biological parameters and compared with some of the known antioxidants. Because of the complex nature of phytochemicals, the antioxidant activities of plant extracts cannot be evaluated by a single method. Therefore, commonly accepted assays, including enzymatic and nonenzymatic methods under *in vitro* and i*n vivo* conditions were employed to evaluate the total antioxidant potential of plant extracts.

DPPH is characterized as stable free radical by virtue of the delocalization of the spare electron, where the molecule as a whole, so that the molecule do not dimerise, as would be the case with most other free radicals. The delocalization gives rise to the deep violet colour, characterized by an absorption band (517 nm). When a solution of DPPH is mixed with a substance of H donor, it gets reduced into nonradical state [37]. Hence, the ethyl acetate extract of *P. hexandrum* exhibited a significant dose-dependant inhibition of DPPH activity. Similar results were reported by [38], while investigating the DPPH radical scavenging activity of wormwood (*Artemisia absinthium* L).

Similarly, results were also reported by [37] that extracts of *Pterocarpus santalinus *exhibited significant DPPH radical scavenging activity. The observed scavenging effect of *P. hexandrum* ethyl acetate extract and standards on the DPPH radical decreases in the following order: *α*-tocopherol > BHT > Ethyl acetate and it was 89.77%, 87.77%, and 85.77% at concentration of 1000 *μ*g/mL, respectively. 

Our results are in tune with earlier investigations that the methanol extracts of exhibited significant DPPH radical inhibition 

The reducing ability of a compound generally depends on the presence of reductants [39] which have been exhibited antioxidative potential by breaking the free-radical chain, donating a hydrogen atom [40]. Presence of reducers causes the conversion of the Fe^3+^/ferricyanide complex used in this method to the ferrous form. By measuring the formation of Perl's Prussian blue at 700 nm, it is possible to determine the Fe^2+^ concentration. Our results suggest that polyphenolic components within the ethyl acetate extract of *P. hexandrum *play an important role in scavenging of free radicals, and the scavenging activity is increased with the increasing concentration of the plant extract. At the higher concentration (300 *μ*g/mL), the reducing power of ethyl acetate extract of *P. hexandrum *and the known antioxidant BHT was 0.53 ± 0.005 and 0.675 ± 0.05, respectively. Our results showed that *P. hexandrum* rhizome ethyl acetate extract is an electron donor and could react with free radicals, convert them to more stable products, and terminate radical chain reaction. Similar results were reported by Noriham et al. [41], who demonstrated antioxidative activity on *Pimpinella anisum *seed extracts and different types of Malaysian plants, and [42] showed that *P. fascisepala* leaf extract is an electron donor and could react with free radicals and terminate radical chain reaction.

Highly reactive OH^●^ radicals are responsible for the oxidative damage of DNA, lipids, and proteins [43]. Formation of a highly reactive tissue-damaging species like hydroxyl radical is caused by the interaction of iron ions with hydrogen peroxide in biological system [44]. The rhizome of *P. hexandrum* ethyl acetate extract exhibited hydroxyl radical scavenging activity in a dose-dependent manner. At a concentration of 300 *μ*g/mL, the extract shows a maximum inhibitory effect of about 83%, which was comparable to the known antioxidant BHT (86%). These results show that ethyl acetate extract of *P. hexandrum* rhizome can act as effective antioxidants by reacting with free radicals. 

Superoxide anion, which is a reduced form of molecular oxygen, has been implicated in the initiating oxidation reactions associated with aging [45]. Also, it has been implicated in several pathophysiological processes, due to its transformation into more reactive species such as hydroxyl radical that initiate lipid peroxidation. Superoxide has also been observed to directly initiate lipid peroxidation [46]. In the Xanthine/Xanthine oxidase system, superoxide anion derived from dissolved oxygen reduces NBT. Antioxidants are able to inhibit the blue NBT formation [47]. The superoxide radical scavenging activity of *P. hexandrum* ethyl acetate extract, and the standard BHT increased in a dose-dependent manner. At the concentration of 300 *μ*g/mL, the percentage inhibition on superoxide radical observed was 69.05 and 78.70%, respectively. Similar results reported by [48] that different extracts from *Croton celtidifolius *Baill (Euphorbiaceae) showed *in vitro* antioxidant properties through the superoxide scavenger capacity method by the nitro blue tetrazolium (NBT) reduction assay.

The measurement of H_2_O_2_-scavenging activity is one of the useful methods of determining the ability of antioxidants to decrease the level of pro-oxidants such as H_2_O_2_ [49]. It can cross membranes and may slowly oxidize a number of compounds. Hydrogen peroxide itself is not very reactive, but sometimes, it can be toxic to cells because of rise in the hydroxyl radicals in the cells [50]. In our study, the H_2_O_2_-scavenging activity of *P. hexandrum* ethyl acetate extract and the standard BHT increased in a dose-dependent manner. At the concentration of 300 *μ*g/mL, the percentage inhibition on H_2_O_2_ was observed 65.54% and 70.91%, respectively. This ability to scavenge hydrogen peroxide could be an efficient assessment method to evaluate antioxidant property of extract of* P. hexandrum.*

In accordance with the results of the antioxidant effects of the extract of *P. hexandrum* rhizome obtained in *in vitro *assays, examinations of the *in vivo *activity of ethyl acetate extract of *P. hexandrum* was also conducted. 

CCl_4_-induced hepatic injuries are commonly used animal models for the screening of hepatoprotective plant extracts and the magnitude of hepatic damage is assessed by measuring the level of released cytosolic transaminases including ALT and AST in circulation [51]. It is generally thought that CCl_4_ toxicity is due to reactive free radical (CCl_3_^●^), which is generated by its reductive metabolism by hepatic cytochrome P450. The reactive intermediate is believed to cause lipid peroxidation and breakdown of cellular membranes [52]. This present study evaluated the hepatoprotective effects of *P. hexandrum* ethyl acetate extract on CCl_4_-induced liver toxicity. Acute administration of CCl_4_ produced a marked elevation of the serum levels of AST, ALT, and LDH in (Group II) treated animals when compared with that of the control group (Group I). Treatment with *P. hexandrum* ethyl acetate extract at a dose of 20, 30, and 50 mg/kg/day significantly reduced the elevated levels of the enzymes (Table 1). Similarly reported by [53] that the ethyl acetate extract of *Cadaba farinosa* exerts protective action by decreasing CCl_4_-derived free radicals and significantly inhibit the elevated level of serum enzyme activities.

Decreased serum levels of AST, ALT and LDH by the plant extract is an indication of stabilization of plasma membrane as well as repair of hepatic tissue damage caused by CCl_4_. The above changes can be considered as an expression of the functional improvement of hepatocytes, which may be caused by an accelerated regeneration of parenchyma cells. 

The body has an effective mechanism to prevent and neutralize the free radical induced damage. This is accomplished by a set of endogenous antioxidant enzymes, such as SOD, CAT, GPX, GST, and GRD. When the balance between ROS production and antioxidant defenses is lost, “oxidative stress” results, which through a series of events deregulates the cellular functions leading to various pathological conditions [54] Any compound, natural or synthetic, with antioxidant properties might contribute towards the partial or total alleviation of this type of damage. In the present study, elevated level of TBARS in CCl_4_-treated rats indicates excessive formation of free radicals and activation of LP system resulting in hepatic damage. TBARS produced as byproducts of LP that occurs in hydrophobic core of bio-membranes [55]. The significant decline in the concentration of these constituents in the liver tissue of CCl_4_ and ethyl extract administered rats indicates antilipid peroxidative effect of *P. hexandrum.*

GSH is a major nonprotein thiol in living organisms which plays a central role in coordinating the body's antioxidant defense processes. Perturbation of GSH status of a biological system has been reported to lead to serious consequences [54]. Decline in GSH content in the liver of CCl_4_-intoxicated rats, and its dose dependent increased level with the administration of extract reveal antioxidant effect of *P. hexandrum*. SOD, GR, GST, and GP_X_ constitute a mutually supportive team of defense against ROS. SOD is a metalloprotein and is the first enzyme involved in the antioxidant defense by lowering the steady-state level of O_2_^−^. GP_X_ is a selenoenzyme two third of which (in liver) is present in the cytosol and one third in the mitochondria. It catalyses the reaction of hydroperoxides with reduced glutathione to form glutathione disulphide (GSSG) and the reduction product of the hydroperoxide. In our study, decline in the activities of these enzymes in CCl_4_-administered rats revealed that LP and oxidative stress elicited by CCl_4_-intoxication have been nullified due to the effect of *P. hexandrum *ethyl acetate extract. Similar results were reported by [56] who investigated hepatoprotective and antioxidant activity of *Boehmeria nivea*. GTS plays an essential role in liver by eliminating toxic compounds by conjugating them with glutathione. GR is concerned with the maintenance of cellular level of GSH (especially in the reduced state) by effecting fast reduction of oxidized glutathione to reduced form. In our study, the liver tissues of CCl_4_-administered rats, activities of GTS and GR were significantly decreased compared with control group. However, pretreatment with ethyl acetate extract of *P. hexandrum,* the activity of these enzymes significantly increased, thus unearthing the antioxidant effect of *P. hexandrum*. Similar results were reported by [57] showed that *Nigella sativa *L and *Urtica dioica* L increase the antioxidant defense system activity in experimentally CCl_4_-treated rats. 

## 6. Conclusion

In conclusion, it is well known that the hepatoprotective effect has a good correlation with the antioxidant activities [58, 59]. In our previous studies, methanolic and 70% ethanolic rhizome extracts of *P. hexandrum* have been demonstrated to possess excellent antioxidant activities by various *in vitro *and *in vivo* assays [5, 6, 60]. In the current study, the encouraging results of the ethyl acetate extract of *P. hexandrum* with the various *in vitro* antioxidant tests proved that the plant possess components with reducing activity has hydrogen-donating ability as well as effectiveness as scavengers of hydroxyl radical, hydrogen peroxide radical, and superoxide free radicals. Current study also demonstrates that ethyl acetate extract could reduce CCl_4_-induced toxicity, particularly hepatotoxicity, by inhibiting lipid peroxidation, suppressing alanine aminotransferase (ALT), aspartate aminotransferase (AST) activities, and lactate dehydrogenase activity (LDH), and increasing antioxidant enzyme activity. Therefore, ethyl acetate extract of *P. hexandrum* can be proposed to protect the liver against CCl_4_-induced oxidative damage in rats, and the hepatoprotective effect might be correlated with its antioxidant and free radical scavenger effects. The studies are in progress to isolate and purify the active principle involved in antioxidant activities of this plant. Further research is needed to study the mechanisms of activity of the active compounds responsible for antihepatotoxic activity.

## Figures and Tables

**Table 1:** Effect of *P. hexandrum* ethyl acetate extract on biochemical parameters in CCl_4_ induced hepatotoxicity in albino rats.

Treatment and groups	Dose (mg/kg)	AST (U/L)	ALT (U/L)	LDH (U/L)
1. Control group (olive oil only)	5 mL/Kg	37.98 ± 3.36	28.06 ± 2.05	89.68 ± 3.54
2. CCl_4_-treated group	1 mL/Kg	118.39 ± 2.83	94.76 ± 2.78	218.62 ± 8.20
3. CCl_4_-treated + V.E	50 mg/Kg	67.55 ± 2.18^$#^	66.58 ± 2.54^$#^	170.88 ± 5.18^$#^
4. CCl_4_-treated + *P.H* extract	20 mg/Kg	100.41 ± 5.40^$#@^	79.33 ± 5.76^$#@^	193.23 ± 5.02^$#@^
5. CCl_4_-treated + *P. H *extract	30 mg/kg	65.37 ± 2.0^$#NS^	67.22 ± 1.91^$#NS^	178.10 ± 4.61^$#b^
6. CCl_4_-treated + *P. H* extract	50 mg/kg	54.16 ± 2.96^$#@^	60.03 ± 2.73^$#@^	168.50 ± 7.20^$#b^

Each value represents the mean ± S.D. of 6 animals. ^$^; *P* < .001, as compared with normal control group, ^#^; *P* < .001 as compared with CCl_4_ group, ^@^; *P* < .001 as compared with V.E, NS; nonsignificant as compared with V.E, b; does not test as compared with V.E. The data were presented as means ± S.D. of six parallel measures and evaluated by one way ANOVA followed by the Bonferroni *t*-test to detect inter group differences. Differences were considered to be statistically significant if *P* < .05.

**Table 2:** Effect of treatment of ethyl acetate extract of *P. hexandrum* on glutathione and antioxidant enzymes in CCl_4_ challenged rats.

Parameters	Group I (olive oil only)	Group II CCl_4_ treated	Group III CCl_4_ + V.E	Group IV CCl_4_ + 20 mg/kg extract	Group V CCl_4_ + 30 mg/kg extract	Group VI CCl_4_ + 50 mg/kg extract
Reduced glutathione (nm/g protein)	103.94 ± 6.03	25.14 ± 2.59^$^	87.40 ± 1.54^$#^	56.65 ± 3.40^$#@^	77.60 ± 1.85^$#@^	85.24 ± 3.87^$#NS^
Glutathione reductase (*μ*g GSSG utilized/minute/mg protein)	32.59 ± 2.10	2.30 ± 0.30^$^	25.44 ± 3.06^$#^	15.36 ± 1.47^$#@^	19.65 ± 1.00^$#@^	24.55 ± 1.22^$#NS^
Glutathione peroxidase (*μ*g GSH utilized/minute/mg protein)	35.48 ± 2.42	3.20 ± 0.77^$^	28.57 ± 1.54^$#^	11.75 ± 1.30^$#@^	18.17 ± 0.31^$#@^	28.57 ± 1.54^$#NS^
Superoxide dismutase (units/mg protein)	35.41 ± 0.65	17.16 ± 1.50^$^	27.75 ± 1.57^$#^	24.96 ± 0.54^$#@^	27.81 ± 0.94^$#b^	28.40 ± 2.47^$#NS^
Glutathione-S-transferase (nmoles of CDNB conjugated/min/mg protein)	15.22 ± 0.69	4.98 ± 0.49^$^	11.94 ± 1.13^$#^	7.60 ± 1.06^$#@^	9.95 ± 0.43^$#@^	12.70 ± 0.57^$#NS^

Each value represents the mean ± S.D. of 6 animals. ^$^; *P* < .001, as compared with normal control group, ^#^; *P* < .001 as compared with CCl_4_ group, ^@^; *P* < .001 as compared with V.E, NS; nonsignificant as compared with V.E, b; does not test as compared with V.E. The data were presented as means ± S.D. of six parallel measures and evaluated by one way ANOVA followed by the Bonferroni *t*-test to detect inter group differences. Differences were considered to be statistically significant if *P* < .05.
